# A psychometric analysis of the reading the mind in the eyes test: toward a brief form for research and applied settings

**DOI:** 10.3389/fpsyg.2015.01503

**Published:** 2015-10-06

**Authors:** Sally Olderbak, Oliver Wilhelm, Gabriel Olaru, Mattis Geiger, Meghan W. Brenneman, Richard D. Roberts

**Affiliations:** ^1^Institute for Psychology and Pedagogy, Ulm UniversityUlm, Germany; ^2^Educational Testing ServicePhiladelphia, PA, USA; ^3^Professional Examination ServiceNew York City, NY, USA

**Keywords:** reading the mind in the eyes test, theory of mind, psychometric analysis, emotion perception, vocabulary, cognitive empathy, short-form

## Abstract

The Reading the Mind in the Eyes Test is a popular measure of individual differences in Theory of Mind that is often applied in the assessment of particular clinical populations (primarily, individuals on the autism spectrum). However, little is known about the test's psychometric properties, including factor structure, internal consistency, and convergent validity evidence. We present a psychometric analysis of the test followed by an evaluation of other empirically proposed and statistically identified structures. We identified, and cross-validated in a second sample, an adequate short-form solution that is homogeneous with adequate internal consistency, and is moderately related to Cognitive Empathy, Emotion Perception, and strongly related to Vocabulary. We recommend the use of this short-form solution in normal adults as a more precise measure over the original version. Future revisions of the test should seek to reduce the test's reliance on one's vocabulary and evaluate the short-form structure in clinical populations.

## Introduction

“Who are you going to believe, me or your own eyes?” – Groucho Marx

The Reading the Mind in the Eyes Test is a popular measure of individual differences in Theory of Mind capabilities (ToM; Baron-Cohen et al., 2001). The test distinguishes many clinical populations (most often individuals on the autistic spectrum) from unimpaired control participants in their ToM capabilities. Despite widespread use of the Reading the Mind in the Eyes Test (hereafter referred to as the Eyes Test, according to a convention established by its authors), little is known about the test's psychometric properties, including the test's factor structure, internal consistency, convergent validity evidence, and the extent to which the measure operates as an effective individual difference measure in normal adults (where it has become increasingly used, e.g., Baron-Cohen et al., 2005). In addition, with the widespread use of this measure in clinical settings, where testing time is always limited, the creation of a short-form version would appear desirable.

In this paper, we present a psychometric analysis of the Eyes Test. First, we present a discussion of ToM and how the Eyes Test was designed to measure this construct, followed by a review of existing estimates of the test's psychometric properties. Then, we present a psychometric evaluation of the complete Eyes Test, followed by an evaluation of other proposed structures (e.g., positive and negative affect subscales). Next, using data driven methods, we attempt to identify possible short-form variants that either maintain or are an improvement over the Eyes Test on particular psychometric parameters. All solutions that show adequate psychometric properties are then related at the latent level with measures of Cognitive Empathy, Emotion Perception, and Vocabulary to estimate the test's convergent validity. All solutions that meet our psychometric criteria are then cross-validated in a second sample. Finally, based on our results, we discuss suggested revisions to the measure.

## Theory of mind

ToM refers to the ability to infer the mental states of others, including intention and knowledge. ToM is referred to as a theory because the inferred mental states (i.e., the mind) are not directly observable and instead one must generate a theory with predictions about how others will behave. ToM was originally developed to describe behavior by chimpanzees (Premack and Woodruff, 1978), and was subsequently extended to describe the development of children in their ability to take the perspective of another person (e.g., Wellman et al., 2001). Thereafter, ToM has been applied to describe social and communicative deficits in specific clinical populations, most commonly individuals on the autism spectrum (e.g., Baron-Cohen et al., 1985). ToM is often considered to be conceptually similar to, or equivalent with, cognitive empathy because both constructs involve inferring the mental state of another person (e.g., Lawrence et al., 2004).

While ToM is typically considered a single, general construct, others have described ToM as representing a multifaceted construct that takes “into account an interconnected network of mental states,” which are described as “perception, attention, belief, desire, intention, emotion, and more” (Astington, 2003, p. 14). As such, Slaughter and Repacholi (2003) argue that if ToM is multifaceted, tests of ToM should also be multifaceted. For example, ToM ability might differ depending on the modality of expression (e.g., voice, face, body) or on actions in that modality (e.g., static representation, moving representation). However, often-used tests of ToM are not deemed multifaceted or multidimensional by their authors, including the Eyes Test (Baron-Cohen et al., 2001).

ToM is theorized to rely heavily on the gaze direction of the person being observed (e.g., Baron-Cohen et al., 2001), which is considered important for social communication and interaction (e.g., Emery, 2000). Many suggest that there is an innate evolved neural system devoted to processing the eye gaze of others; this is supported by experimental research with infants demonstrating that infants as young as several hours old prefer faces with open eyes (Batki et al., 2000). Moreover, research with adults suggests that this population has difficulty ignoring the eye gaze of others (Posner, 1980), with functional imaging research suggesting that the posterior superior temporal sulcus and areas of the medial frontal cortex are involved in both eye gaze and ToM tasks (Calder et al., 2002).

Along this line, ToM is related to the ability to perceive emotions expressed in the face. In particular, the Eyes Test has been shown to be moderately to strongly related with measures of emotion perception ability (Henry et al., 2009; Petroni et al., 2011). Indeed, in some cases the Eyes Test has been used as a measure of emotion perception (e.g., Guastella et al., 2010).

ToM is also associated with language ability. Research suggests there is a developmental threshold around a particular level of language development, and before this threshold, children cannot successfully “pass” any ToM measure (Jenkins and Astington, 1996). For example, one longitudinal study found that, when controlling for earlier ToM performance, language ability (syntax and semantics) predicted current ToM, but earlier ToM did not predict current language abilities (Astington and Jenkins, 1999). As a result, some researchers have suggested that it is specific aspects of verbal ability that predict performance on ToM measures (Lawrence et al., 2004; Peterson and Miller, 2012). In general, verbal abilities are associated with performance on the Eyes Test (Golan et al., 2006; Ahmed and Miller, 2011; Peterson and Miller, 2012; cf. Ferguson and Austin, 2010 who found no relation).

## Psychometric evaluation of the eyes test

The Eyes Test was designed to measure the first stage of ToM attribution, which is identifying the relevant mental state of the stimulus (as opposed to the second stage; inferring the content of that mental state; see Baron-Cohen et al., 2001). Accordingly, the Eyes Test was developed under the premise that ToM is heavily based on the perception of eye gaze in others. The current assessment (i.e., Version 2) includes 36 items where participants view the eyes of a person and must select which of four terms best describes the intention of a target person. Of note, this version of the Eyes Test is considered superior to earlier versions, largely on the grounds of reliability and validity evidence (see Baron-Cohen et al., 2001). The Eyes Test has been translated into a variety of languages including: Bosnian (Schmidt and Zachariae, 2009), French (Prevost et al., 2014), Greek (child version; Vogindroukas et al., 2014), Italian (Vellante et al., 2012), Japanese (Kunihira et al., 2006; Adams et al., 2009), Persian (Khorashad et al., 2015), Romanian (Miu et al., 2012), Spanish (Fernández-Abascal et al., 2013), Swedish (Hallerbäck et al., 2009), and Turkish (Girli, 2014).

### Reliability

Based on published estimates, the Eyes Test typically has poor internal consistency (Voracek and Dressler, 2006; Harkness et al., 2010; Ragsdale and Foley, 2011; Vellante et al., 2012; Khorashad et al., 2015; cf. Dehning et al., 2012; Girli, 2014; Prevost et al., 2014, for an exception). The Eyes Test also does not meet assumptions of normality (Söderstrand and Almkvist, 2012; Vellante et al., 2012). However, the test-retest reliability of the measure is acceptable (Hallerbäck et al., 2009; Yildirim et al., 2011; Vellante et al., 2012; Prevost et al., 2014; Khorashad et al., 2015). The low internal consistency estimates may be a function of various test attributes. For example, there are many inconsistencies between the items (e.g., commonness of response option words, uneven presentation of identities, angle of face), which may reduce the internal consistency of the test. In addition, there is limited standardization of the picture characteristics (e.g., ratio of dark and light, use of shadows, artifacts present), which may be a confound. To this end, Hallerbäck et al. (2009) found that changing the lighting of one image increased the performance on that item.

It seems plausible that the low internal consistency occurs because the test does not have a single factor solution, but instead measures several factors. While the Eyes Test is proposed to measure a single construct (Baron-Cohen et al., 2001), as noted previously others have suggested that there might be subscales composed of those items that represent positive, negative, and neutral affect (Harkness et al., 2005; Maurage et al., 2011; Konrath et al., 2014). However, the results of a confirmatory factor analysis (CFA) suggest that the division of items into an affect specific factor is a poor fit to the data (Vellante et al., 2012). Whether this result is replicable across various populations remains uncertain.

In summary, available research with the Eyes Test suggests that in general, the test has poor internal consistency and there is limited evidence of the test's homogeneity. This study will re-examine these assertions based on two samples of Amazon Mechanical Turk workers, who appear more representative of the general population than are the typical first year psychology students comprising many of the studies reviewed in the preceding (see e.g., Berinsky et al., 2012).

### Validity evidence

The results of several studies suggest that the Eyes Test scores show convergent validity evidence with other ToM measures, specifically scores from the Strange Stories Test and the Faux Pas Test (Torralva et al., 2009; Ferguson and Austin, 2010; Kirkland et al., 2012a), however, others have found no relation (Ahmed and Miller, 2011; Duval et al., 2011). Likewise, the relation between the Eyes Test and self-report measures of Cognitive Empathy is mixed; the test has been shown to be both weakly negatively related (Spreng et al., 2009) and weakly positively related to self-reported cognitive empathy (Grove et al., 2014).

However, the test can successfully differentiate between groups presumed to differ in their ToM abilities, specifically between groups with and without autism or Asperger's syndrome (e.g., Baron-Cohen et al., 1985, 1997). In addition, researchers have found schizophrenic patients (Bora et al., 2009) and alcoholics (Maurage et al., 2011) perform worse on the Eyes Test compared to controls.

## The current studies: Rationale

Despite its widespread use, little is known about the psychometric properties of the Eyes Test. Several studies identified the test as having poor internal consistency and some have proposed that the test is not unidimensional but instead measures multiple factors. Given that this test is frequently applied in clinical settings, the poor internal consistency in particular is troubling, because it suggests it is unclear what exactly is being measured by the current version of the test. A reduced scale would be beneficial for the assessment field because it would be quicker to administer and if the steps taken to shorten the measure focus on increasing the test's homogeneity, the short-form version would also be a more precise measure of ToM.

The purpose of the current study was to perform a rigorous psychometric evaluation of the Eyes Test. First, we test the adequacy of the original version - a single-factor solution including all items—and assess the extent to which the test is internally consistent (i.e., all items are related to one another) and homogenous (i.e., unidimensional with the items measuring a single latent trait; Clark and Watson, 1995). In addition, we test various short-form versions and subscales proposed in the literature, followed by the application of two data driven methods with the goal of identifying a short-form solution, while simultaneously improving the test's precision.

Each test version (i.e., the full scale, short-form solutions, individual subscales) will be evaluated on the extent to which that test version satisfies two psychometric criteria: (1) measurement model fit, including adequate factor loadings, in a CFA; (2) adequate omega estimate, which is based on the results of the CFA and indicates the test's factor saturation or “the reliability of a test score …the precision with which a homogeneous test measures the common attribute of its items” (McDonald, 1999, p. 90). We chose these two tools, instead of traditional measures of internal consistency (i.e., Cronbach's Alpha [Cronbach, 1951] or KR-20 for dichotomous data [Kuder and Richardson, 1937]) because unlike traditional estimates which do not assess homogeneity (Cortina, 1993; Clark and Watson, 1995), omega and CFA, simultaneously provide estimates of both. Also, Cronbach's alpha is highly biased by long tests; Cortina (1993) recommended Cronbach's alpha not be used for tests with more than 40 items and the Eyes Test, at 36 items, is close to this limit.

Those scale solutions that meet both of our psychometric criteria will be correlated at the latent-level with measures of Emotion Perception, Cognitive Empathy, and Vocabulary. Given previous research reviewed above, we expect moderate relations with all three constructs. Finally, we will cross-validate those scale solutions that adequately meet our criteria in a second sample.

## Study 1

### Methods

#### Participants

Participants were recruited through Amazon Mechanical Turk, an online survey website and provided $8.00 for participating. We chose Mechanical Turk because research suggests this platform can be used to obtain data from a diverse sample and the data is comparable to samples collected with traditional methods (Buhrmester et al., 2011; Casler et al., 2013). Initially, 616 individuals participated; 127 were removed because they failed to correctly respond to at least one attention check questions (an admittedly strict criteria, designed to ensure compliant respondents). An additional three people were removed because there was no variance in their response across an entire scale (i.e., the person consistently selected the same response). Finally, we identified two outliers in the Eyes Test, where participants performed substantially worse than the rest of the sample. When those two persons were removed, the Eyes Test composite scores were more normally distributed, so these two persons were removed for the remainder of the analyses. Our final sample consisted of 484 participants (243 female), 33.4 years of age (*SD* = 11.0), primarily White, non-Hispanic (76%; 7% Black, non-Hispanic; 8% Asian; 9% Other), from a variety of education levels (13% High School or GED equivalent, 44% Some College, 43% Bachelors Degree or higher). All participants were currently living in America (indicated by their IP addresses), with 95% born in America, and for 96% their native language was English.

#### Measures

Participants completed several measures online (measures presented at http://www.unipark.de/); as part of a larger study examining various measures of cross-cultural competence, inclusive of predictors, moderators, and mediators. In the passages that follow, we only discuss those measures considered relevant to our proposed research questions.

##### Reading the mind in the eyes

The Eyes Test is a 36-item measure originally developed to measure ToM in adults (Baron-Cohen et al., 2001). The current version (Version 2) uses four response options consisting of the correct target word and three incorrect foil words. Each item is scored as correct or incorrect.

##### Perspective taking

The Perspective Taking subscale of the Interpersonal Reactivity Index (Davis, 1980) is a 7-item questionnaire that is considered a measure of Cognitive Empathy. An example item is “I try to look at everybody's side of a disagreement before I make a decision” and the response options range from 1 “Does not describe my well” to 5 “Describes me well.” In our sample, the factor saturation was excellent (ω = 0.93).

##### Emotion specific empathy cognitive empathy

The Cognitive Empathy subscale of the Emotion Specific Empathy questionnaire (ESE; Olderbak et al., 2014) is a 30-item measure that assesses Cognitive Empathy. An example item is “It is easy for me to understand why others become sad when something heartbreaking happens to them” and the response options range from −3 “Strongly Disagree” to 3 “Strongly Agree.” In our sample, factor saturation for the Cognitive Empathy subscale was excellent (ω = 0.95)

##### Diagnostic analysis of non-verbal accuracy version 2 – faces (DANVA 2)

The DANVA 2 Faces subscale (Nowicki and Duke, 1994) is a 24-item measure that assesses the ability to perceive emotions in the face. The test presents emotional faces with limited presentation time, four response options (happy, sad, angry, fearful), and is scored as correct or incorrect. Factor saturation was excellent (ω = 0.93).

##### Vocabulary

This is a 4-choice synonym vocabulary test consisting of 18 items developed by the Educational Testing Service and items are scored as correct or incorrect (Ekstrom et al., 1976). Each item presents a target word (e.g., handicraft) and participants must select among the response options best describes the target word (e.g., cunning, fast boat, utility, manual skill, and guild). Factor saturation was acceptable (ω = 0.79).

#### Procedure

Each person was tested online, un-proctored as a part of a larger study examining cross cultural competence. At the end of the testing sessions, participants were reimbursed for their time. All tests and protocols were approved by the Educational Testing Service Human Ethics and Fairness Review Committee.

### Results

Because the data are dichotomous items indicative of a latent trait, we chose to work with a tetrachoric correlation matrix (estimated with PROC FREQ in SAS 9.3).

#### Full scale eyes test analysis

##### Descriptive statistics

The data were normally distributed (skew = −0.51; kurtosis = −0.06) with participants, on average, receiving high scores (*M* = 27.20, *SD* = 3.82, Average percent correct = 76%), with individual level scores ranging from 16 (44% correct) to 36 (100% correct). With the exception of item 17, the most frequently chosen response option was the correct response option (see Table A1 in Supplementary Materials), which is supported by prior studies of normal populations (e.g., Baron-Cohen et al., 2001; Harkness et al., 2005). The sample-level scores are similar to other studies with participants on average correctly answered over half of the items (e.g., in Baron-Cohen et al. (2001) the sample-level scores for normal populations were high, ranging from 26.2 to 30.9 [74–86% correct] and for individuals with Asperger's syndrome or High Functioning Autism the average score was lower at 21.9 [61% correct]).

There was no relation between the Eyes Test and age (*r* = 0.08, *p* = 0.07) or education [*F*_(4, 479)_ = 1.22, *p* = 0.30^1^; see Söderstrand and Almkvist (2012), for similar results with education]. Theory suggests females are higher in empathy than males (Baron-Cohen et al., 2005) and thus will perform better on the Eyes Test; however, this was also not supported in our study [*t*_(481)_ = 1.29, *p* = 0.20^2^, see Kirkland et al., 2012b; Vellante et al., 2012; but cf. Söderstrand and Almkvist, 2012].

##### Inter-item correlations

**Figure 1:**
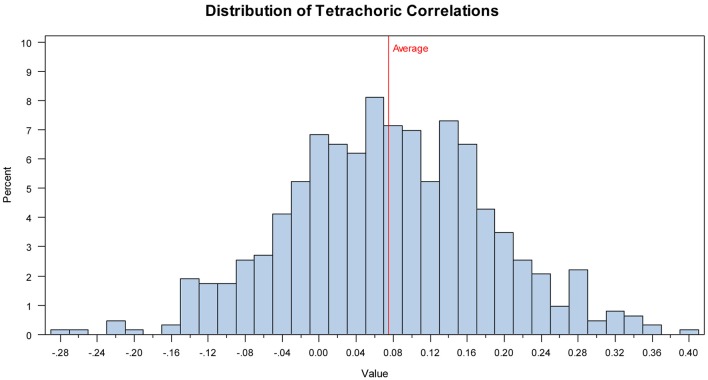
**Study 1: Distribution of the tetrachoric correlations**.

The tetrachoric correlations between items ranged from −0.28–0.41, with average inter-item correlation at 0.08, suggesting there is weak agreement between items, with many items negatively related to one another (interestingly, relations between those items that shared the same target word were also weak [Cautious; *r* = 0.08; Fantasizing: *r* = 0.28; Preoccupied: *r* = 0.16]). The average inter-item correlation, as well as the range of inter-item correlations, are outside the range recommended by Clark and Watson (1995) for sufficient internal consistency (recommended values range from 0.15 to 0.50). This pattern of correlations suggest that more than one factor might underlie the Eyes Test (Piedmont and Hyland, 1993; see Figure 1 for the distribution of correlations and Table A2 in Supplementary Materials for the full correlation matrix).

##### Exploratory factor analysis

Because others have suggested there are multiple factors assessed by the Eyes Test, and because many of the inter-item correlations are negative, again suggesting more than one factor, we performed an exploratory factor analysis (EFA) to assess the number of factors present in the data. Because the data are dichotomous, we applied the estimator robust weighted least squares means and variance adjusted (WLSMV; Muthén and Muthén, 2011) on the raw data, which is most frequently used with large sample sizes and dichotomous data (Flora and Curran, 2004; Beauducel and Herzberg, 2006); this method estimates standard errors so we can apply significance testing to the overall factor structure. A geomin (i.e., oblique) rotation was used so that factors were allowed to correlate (however this does not prevent an orthogonal structure from still being identified). Beginning with a two-factor solution, we increased the number of identifiable factors until, according to the model's fit indices, the structure showed adequate fit to the data (see Table 1). According to all fit indices the five-factor solution was the best fit to the data. However, an examination of the factor pattern, identifying items with moderate loadings or higher (>0.30), indicated that only 27 of the 36 items moderately loaded on at least one factor, with several items unrelated to any of the five factors (Table 2). Also, the factors were either weakly or unrelated with one another (see Table 3). Given that the Eyes Test is purported to measure a single factor, and that none of these five factors was previously postulated, we instead applied CFA to examine the factor structure of the test.

**Table 1:** **Study 1: Exploratory factor analysis fit indices for the two to five-factor solutions**.

	**Chi-square**	**RMSEA**	**CFI**	**TLI**
2 Factors	χ^2^_(559)_ = 623.02, *p* < 0.05	0.015 _(0.005–0.022)_	0.846	0.826
3 Factors	χ^2^_(525)_ = 564.01, *p* = 0.12	0.012 _(0.000–0.020)_	0.906	0.887
4 Factors	χ^2^_(492)_ = 525.30, *p* = 0.14	0.012 _(0.000–0.020)_	0.920	0.897
5 Factors	χ^2^_(460)_ = 488.45, *p* = 0.17	0.011 _(0.000–0.020)_	0.931	0.906

**Table 2:** **Study 1: Exploratory factor analysis: five-factor solution with rotated factor loadings of eyes test items**.

**Item**	**Target Word**	**Factor 1**	**Factor 2**	**Factor 3**	**Factor 4**	**Factor 5**
3	Desire	**0.703**	−0.026	0.066	−0.216	0.214
30	Flirtatious	**0.500**	0.038	−0.120	−0.087	−0.028
21	Fantasizing	**0.419**	−0.140	0.007	**0.353**	−0.003
6	Fantasizing	**0.391**	−0.005	−0.024	0.286	−0.200
25	Interested	**0.358**	**0.318**	−0.248	0.012	−0.054
34	Distrustful	**0.317**	−0.023	**0.428**	0.013	0.008
9	Preoccupied	−0.042	**0.640**	−0.026	−0.039	−0.136
19	Tentative	0.007	**0.509**	0.246	−0.357	−0.026
14	Accusing	−0.018	**0.481**	0.061	−0.010	0.099
36	Suspicious	0.170	**0.443**	−0.049	−0.346	0.181
8	Despondent	−0.048	**0.368**	**0.351**	0.083	0.094
16	Thoughtful	−0.166	**0.329**	0.128	0.081	−0.025
10	Cautious	−0.004	−0.003	**0.550**	−0.046	−0.060
24	Pensive	0.127	0.171	**0.450**	0.004	−0.045
5	Worried	0.259	0.075	**0.420**	0.032	−0.318
7	Uneasy	−0.057	−0.093	**0.405**	−0.052	0.139
4	Insisting	−0.034	−0.111	**0.326**	−0.014	0.230
35	Nervous	0.222	0.024	**0.321**	−0.043	0.221
20	Friendly	−0.054	0.144	0.067	**0.436**	−0.045
1	Playful	0.018	0.098	−0.076	**0.339**	0.156
23	Defiant	−0.003	0.050	−0.015	−0.091	**0.565**
28	Interested	−0.004	0.197	0.040	0.152	**0.414**
22	Preoccupied	0.050	0.174	−0.008	0.214	**0.347**
2	Upset	0.273	0.009	0.029	0.216	0.033
11	Regretful	0.268	0.032	0.135	0.098	0.146
12	Skeptical	−0.144	0.264	0.168	−0.046	0.050
13	Anticipating	0.132	0.189	−0.130	0.110	0.133
15	Contemplative	0.008	0.296	0.211	0.066	0.082
17	Doubtful	−0.257	0.006	0.166	0.025	0.230
18	Decisive	0.010	−0.034	0.221	0.233	0.254
26	Hostile	0.100	−0.032	0.124	0.076	0.024
27	Cautious	0.028	0.094	0.069	0.055	0.274
29	Reflective	0.145	0.177	−0.038	0.107	−0.008
31	Confident	0.125	0.134	0.027	0.112	−0.190
32	Serious	0.025	0.275	0.131	0.240	−0.020
33	Concerned	−0.019	0.289	−0.048	0.272	0.119

*In the above table, all loadings above 0.30 are bolded*.

**Table 3:** **Study 1: Exploratory factor analysis inter-factor correlations for the five-factor solution**.

	**Factor 1**	**Factor 2**	**Factor 3**	**Factor 4**	**Factor 5**
Factor 2	0.122				
Factor 3	0.019	0.140			
Factor 4	0.021	0.214^*^	0.105		
Factor 5	0.025	0.146	0.233	−0.008	

* *p < 0.05*.

##### Measurement model

The full version of the Eyes Test was modeled in CFA using WLSMV. Since the authors of the Eyes Test propose that all items are indicators of a single construct the test was modeled such that each item loaded on a single latent variable (see Table 4). While some might argue against using individual items as indicators (e.g., Nunnally and Bernstein, 1994), and instead advocate for parceling, a requirement for parceling is knowing the factor structure supporting each item (Little et al., 2002). Since there is no suggestion as how to create the parcels, that method was not employed.

**Table 4:** **Study 1: confirmatory factor analysis results, including factor loadings (β) and variance explained, for a 1-factor solution**.

**Item**	**Target word**	**Fully standardized betas (β)**	**Indicator variance explained**
1	Playful	0.208^*^	0.043
2	Upset	0.197^*^	0.039
3	Desire	0.280^*^	0.078
4	Insisting	0.252^*^	0.064
5	Worried	0.253^*^	0.064
6	Fantasizing	0.086	0.007
7	Uneasy	0.249^*^	0.062
8	Despondent	0.560^*^	0.314
9	Preoccupied	0.326^*^	0.106
10	Cautious	0.310^*^	0.096
11	Regretful	0.307^*^	0.094
12	Skeptical	0.271^*^	0.073
13	Anticipating	0.190^*^	0.036
14	Accusing	0.418^*^	0.175
15	Contemplative	0.421^*^	0.177
16	Thoughtful	0.275^*^	0.076
17	Doubtful	0.164^*^	0.027
18	Decisive	0.338^*^	0.114
19	Tentative	0.367^*^	0.135
20	Friendly	0.242^*^	0.059
21	Fantasizing	0.152	0.023
22	Preoccupied	0.384^*^	0.147
23	Defiant	0.284^*^	0.081
24	Pensive	0.439^*^	0.193
25	Interested	0.127^*^	0.016
26	Hostile	0.133^*^	0.018
27	Cautious	0.289^*^	0.084
28	Interested	0.428^*^	0.183
29	Reflective	0.170^*^	0.029
30	Flirtatious	0.073	0.005
31	Confident	0.080	0.006
32	Serious	0.351^*^	0.123
33	Concerned	0.307^*^	0.094
34	Distrustful	0.371^*^	0.138
35	Nervous	0.406^*^	0.165
36	Suspicious	0.313^*^	0.098

* *p < 0.05*;

*Light shaded cells indicates a weak relation (β < 0.30), medium shaded cells indicate a moderate relation (0.30 ≥ β < 0.50), and dark shaded cells indicate a strong relation (β ≥ 0.50)*.

Model fit was evaluated according to established standards, specifically RMSEA < 0.06, CFI ≥ 0.95, TLI ≥ 0.95 (Hu and Bentler, 1999), and WRMR < 0.90 (Yu, 2002). Based on several indicators model fit was poor [$\chi_{\left(594,\;n=484\right)}^{2}=707.03$, *p* < 0.05; *RMSEA* = 0.020_(0.013–0.025)_; *WRMR* = 1.042; *CFI* = 0.728; *TLI* = 0.711]. Specifically, the model fit of a single factor was not acceptable according to the χ^2^ goodness-of-fit statistic. However, while χ^2^ is considered an important statistic for interpretation, it is overly influenced by sample size and thus models can be easily rejected. The absolute fit index RMSEA (Steiger and Lind, 1980), which estimates the amount of misfit per degree of freedom by adjusting the chi-square measure to account for degrees of freedom, thus ceteris paribus simple models are preferred, indicated good fit. However, the RMSEA will result in artificially lower estimates when there are weak factor loadings (Heene et al., 2011; Savalei, 2012), as is the case in this model, which can lead to an acceptance of a larger saturated model (Rigdon, 1996). Finally, model fit according to WRMR (Yu, 2002), and the relative or incremental fit indices CFI (Bentler, 1990) and TLI (Tucker and Lewis, 1973), which are based on the percent of improvement compared to the null model, model fit was poor (e.g., Kline, 2005).

A single factor was also a poor predictor of a majority of the items. Twenty items (56% of the scale) had weak loadings with the average loading at 0.278 (loadings ranged from 0.073 to 0.560). For all items there was more residual variance (average = 0.910) than variance explained by the single factor (average = 0.090), with the single factor not explaining more than 31% of the variance for any item. These results are not surprising, given the low inter-item correlations, and suggest that a single factor solution for all 36 items is not a sufficient representative fit to the data.

##### Omega

Next, based on the factor loadings in the CFA we estimated ordinal omega (ω; equation 6.20 b in McDonald, 1999), which is the traditional omega based on the tetrachoric correlation matrix, and provides an estimate of factor saturation and internal consistency. According to omega the factor saturation and internal consistency of the scale is acceptable (ω = 0.75). However, given that many of the items are unrelated to one another, and in many instances, negatively related, this estimate is most likely inflated because of the test's length (Nunnally and Bernstein, 1994; Zinbarg et al., 2006).

##### Convergent validity evidence

Convergent validity evidence was estimated in a CFA by correlating the full Eyes Test, modeled as a latent variable indicated by its 36 items, with a measure of Cognitive Empathy, Emotion Perception, or Vocabulary, where each construct was also modeled as a latent variable, indicated by either individual items (Perspective Taking), parcels (Vocabulary), or emotion-specific subscales (ESE Cognitive Empathy and the DANVA; see Table 7). However, given the poor fit of the single factor solution to the data in a CFA, the fit of these models is poor, thus the correlations could not be meaningfully interpreted.

##### Conclusion

Overall, based on the presented results, we suggest that the complete Eyes Test does not have a single factor solution. The inter-item correlations are low, with many items unrelated to one another, and in many instances, negatively related, suggesting that the omega estimate may be inflated because of the test's length. Second, an EFA identified five factors, with these factors weakly correlated with one another and many items not loading on any factor. Finally, in a CFA a single factor solution did not adequately fit the data, with large residual variances remaining for a majority of the items. Overall, the test is not homogeneous. To result in superior psychometric properties, the test would most likely benefit from being reduced to a short-form solution with a focus on the test's homogeneity.

### Revising the eyes test structure

Next, we identified and tested several revised versions of the Eyes Test with the goal of addressing the shortcomings identified in the full version. The first set of models are based on structures proposed in the literature, with the last two based on data driven methods. The first model tested is a reduced 17-item version proposed by Konrath et al. (2014). The next two models are based on the valence of the target response options and identify positive, negative, and in one case, neutral affect subscales. The last set of models were identified through data driven methods: (1) Maximizing Main Loadings, and (2) an adaptation of the Ant Colony Optimization method (Marcoulides and Drezner, 2003, adaptation by Olaru et al., in press). Each revised version will be evaluated according to same criteria applied above. In order to compare sample-level scores between the revised scales, the Eyes Test items will be averaged instead of summed.

#### Konrath reduced scale

Konrath et al. (2014) utilized a short-form measure of the Eyes Test, including only 17 of the full 36 items, and reported the internal consistency was poor (α = 0.23). Unfortunately, only the target word of those items was reported, three of which appear twice in the original scale. As mentioned earlier, those three items are weakly correlated with one another, suggesting that in a CFA two items with the same target word are not interchangeable. As such, we iteratively tested all combinations of the repeated items with CFA; results for the best set of items, according to the CFA fit indices, are presented in Tables 4–6. In a CFA this structure was an improved fit according to the CFA fit indices when compared with the complete Eyes Test, however only five of the 17 items had moderate or higher loadings on the single factor, and five items were not even significantly related to the central construct, suggesting that single factor did not adequately account for the variance in the 17 items and this solution had poor structural validity evidence. Similarly, the omega estimate of this short-form structure was poor indicating poor factor saturation and internal consistency. Finally, based on the latent correlations, this short-form solution had weak to moderate relations with Cognitive Empathy, moderate relations with Emotion Perception, and was strongly related with Vocabulary. Because this short-form solution has poor internal consistency according to omega and had poor structural validity evidence, this structure is not a sufficient solution.

**Table 5:** **Study 1 and 2: Comparison of possible short-form and subscale solutions**.

**Model**	**Total number of items (Females)**	**Descriptive statistics**				**Omega (ω)**	**CFA—Fit indices**				
		***M***	***SD***	**Skew**	**Kurtosis**		**Chi-square**	**RMSEA _(*CIs*)_**	**WRMR**	**CFI**	**TLI**
**STUDY 1**											
Full Scale	36 (17)	0.76	0.11	−0.51	0.06	0.75	χ^2^_(594)_ = 707.03, *p* < 0.05	0.020 _(0.013–0.025)_	1.042	0.728	0.711
Konrath Model											
Reduced	17 (6)	0.77^*^	0.12	−0.64	0.17	0.58	χ^2^_(119)_ = 126.15, *p* = 0.31	0.011 _(0.000–0.026)_	0.876	0.914	0.902
Positive	6 (2)	0.80^*^	0.17	−0.62	−0.16	na	na				
Negative	9 (3)	0.74	0.15	−0.47	−0.07	na	na				
Harkness Model											
Negative affect	12 (5)	0.72	0.15	−0.33	−0.32	0.55					
Positive affect	8 (5)	0.82	0.15	−0.66	0.01	0.48	χ^2^_(591)_ = 694.77, *p* < 0.05	0.019 _(0.012–0.025)_	1.032	0.750	0.733
Neutral affect	16 (7)	0.75	0.14	−0.66	0.31	0.67					
MML solution	7 (4)	0.69	0.22	−0.57	−0.13	0.64	χ^2^_(14)_ = 21.18, *p* = 0.10	0.033 _(0.000–0.059)_	0.777	0.941	0.912
ACO model	10 (4)	0.81	0.17	−1.22	1.90	0.70	χ^2^_(35)_ = 44.25, *p* = 0.14	0.023 _(0.000–0.042)_	0.826	0.937	0.918
**STUDY 2**											
ACO Model	10 (4)	0.72	0.19	−0.89	1.09	0.73	χ^2^_(35)_ = 34.85, *p* = 0.48	0.000 _(0.000–0.049)_	0.699	1.000	1.002^+^

* *Indicates there is a significant difference between the performance of male and female participants, with females performing better than males. na, statistic could not be estimated*.

+ *, TLI can sometimes fall outside of the 0–1 range (Kline, 2005)*.

**Table 6:** **Study 1 and 2: confirmatory factor analysis fully standardized loadings (β) for all short-form and subscale solutions**.

**No**.	**Target Word**	**Sex**	**Study 1**						**Study 2**
			**Konrath model–reduced**	**Harkness model**			**MML**	**ACO model**	**ACO model**
				**Negative**	**Positive**	**Neutral**			
1	Playful	M	0.155		0.393^*^				
2	Upset	M	0.227^*^	0.205^*^					
3	Desire	F	0.240^*^			0.305^*^			
4	Insisting	M	0.289^*^			0.244^*^			
5	Worried	M	0.173	0.273^*^					
6	Fantasizing	F	0.065		0.261^*^				
7	Uneasy	M	0.144			0.230^*^			
8	Despondent	M	0.678^*^			0.611^*^	0.545^*^	0.653^*^	0.616^*^
9	Preoccupied	F				0.427^*^		0.482^*^	0.510^*^
10	Cautious	M				0.318^*^	0.442^*^		
11	Regretful	M	0.255^*^	0.334^*^					
12	Skeptical	M	0.362^*^			0.297^*^		0.406^*^	0.669^*^
13	Anticipating	M	0.225^*^			0.166^*^			
14	Accusing	M	0.428^*^	0.431^*^				0.476^*^	0.376^*^
15	Contemplative	F	0.468^*^			0.450^*^	0.351^*^	0.437^*^	0.635^*^
16	Thoughtful	M	0.210^*^		0.393^*^				
17	Doubtful	F	0.145	0.164^*^					
18	Decisive	F				0.355^*^			
19	Tentative	F				0.347^*^	0.411^*^	0.413^*^	0.139
20	Friendly	M			0.424^*^				
21	Fantasizing	F			0.314^*^				
22	Preoccupied	F	0.327^*^	0.406^*^				0.353^*^	0.242^*^
23	Defiant	M		0.308^*^					
24	Pensive	M				0.457^*^	0.482^*^	0.390^*^	0.451^*^
25	Interested	F			0.249^*^				
26	Hostile	M		0.139					
27	Cautious	F	0.214^*^	0.297^*^					
28	Interested	F				0.448^*^			
29	Reflective	F				0.189^*^			
30	Flirtatious	F			0.150				
31	Confident	F			0.220^*^				
32	Serious	M				0.397^*^		0.393^*^	0.480^*^
33	Concerned	M				0.349^*^			
34	Distrustful	F		0.400^*^			0.487^*^		
35	Nervous	F		0.444^*^			0.437^*^		
36	Suspicious	M		0.335^*^				0.316^*^	0.454^*^

* p < 0.05

*Shaded cells indicate the item belongs to that particular subscale. Light shaded cells indicates a weak relation (β < 0.30), medium shaded cells indicate a moderate relation (0.30 ≥ β < 0.50), and dark shaded cells indicate a strong relation (β ≥ 0.50)*.

**Table 7:** **Study 1 and 2: correlations of the eyes test with the latent constructs cognitive empathy, vocabulary, and emotion perception**.

	**IRI perspective taking**	**ESE cognitive empathy**	**Emotion perception**	**Vocabulary**
**STUDY 1**				
**Original scale**	**0.11**	0.21^*^	0.35^*^	0.54^*^
**Konrath model–reduced**	0.16^*^	0.34^*^	0.38^*^	0.59^*^
**MML solution**	**0.07**	**0.11**	0.19^*^	0.46^*^
**ACO model**	**0.15**	0.21^*^	0.34^*^	0.49^*^
**STUDY 2**				
**ACO model**	0.25^*^	0.56^*^	0.29^*^	0.62^*^

* *p < 0.05*;

*Light shaded cells indicates a weak relation (r < 0.30), medium shaded cells indicate a moderate relation (0.30 ≥ r < 0.50), and dark shaded cells indicate a strong relation (r ≥ 0.50)*.

#### Konrath affect model

Konrath et al. (2014) also proposed two subscales - (1) Positive Affect; and (2) Negative Affect—with two of the 17 items from the Konrath Reduced Scale Model not used on either subscale. In a single CFA, we modeled both subscales as two latent constructs indicated by their respective items and correlating with one another. The model resulted in an improper solution, with the predicted covariance matrix not positive definite (this also occurred with the factors modeled separately) suggesting this solution was not an adequate fit to the data. As such, omega could not be estimated. Because these subscales could not be modeled in a CFA, their latent correlations with Cognitive Empathy, Vocabulary, and Emotion Perception were not estimated. Overall, this subscale structure is not a sufficient solution.

#### Harkness model

Harkness et al. (2005) also identified three affect-based subscales: (1) Positive Affect; (2) Negative Affect; and (3) Neutral Affect. When modeled in a CFA, with the three subscales modeled as latent factors indicated by their respective items and with the factors correlated with one another, the test structure was a poor fit to the data according to the fit indices, with many items loading weakly on their respective latent factor (note: the poor fit is also supported by (Vellante et al., 2012)). The interfactor correlations were moderate (Negative Affect with Positive Affect: *r* = 0.399, *p* < 0.05; Positive Affect with Neutral Affect: *r* = 0.490, *p* < 0.05), with the correlation between the Negative Affect and Neutral Affect subscales strong (*r* = 0.876, *p* < 0.05), suggesting the latter two subscales measure similar constructs. The omega estimates were poor for all three subscales indicating poor factor saturation and internal consistency. Finally, because the measurement model was a poor fit to the data, the latent correlations with Cognitive Empathy, Vocabulary, and Emotion Perception were not estimated. Overall, these results suggest this short-form structure is not a sufficient solution.

These results suggest that none of the empirically proposed short-form or subscale structures adequately fit the data. Next, we applied two statistical tools in a data-driven fashion to identify the best fitting short-form structure according to that particular statistical tool that maximize one or both of our criteria. In both attempts, we are assuming that all items are equal indicators of ToM.

#### Maximizing main loadings (MML) solution

First, we used CFA to identify the maximum number of items that could be identified by a single latent construct in a model that had adequate fit according to chi-square, RMSEA, CFI, and TLI. First, all 36 items were modeled in a single CFA, with all items loading on a single factor. Then, the item with the weakest loading was removed and the model was re-estimated for the reduced item set; this was done iteratively until the model had adequate fit according to all fit indices. This process resulted in a much reduced model, retaining only seven of the original 36 items. This final model showed acceptable fit according to the CFA fit indices, with all items moderately or strongly loading on the latent factor. At the latent level, the MML Solution was not significantly related to Cognitive Empathy, weakly related to Emotion Perception, and moderately related to Vocabulary. However, despite an adequate measurement model structure, this short-form solution has a poor omega estimate indicating poor internal consistency and factor saturation, so this short-form solution was also not sufficient.

#### Ant colony optimization

ACO is a heuristic algorithm that converges to an optimal or close-to-optimal solution over the course of iterations. The criterion to be optimized can be specified freely, for example, maximizing CFI, minimizing RMSEA, maximizing standardized loadings, etc. Subsets of items are picked based on probabilities, and these probabilities are then modified after each iteration based on the suitability of each item to reach the specified criterion. Assuming an item contributes to improving the specified criterion, ACO will then increase the probability of that item in the subsequent subset of items (Leite et al., 2008). In a study comparing ACO with other methods to identify a short-form solution, the ACO method was found to be an efficient procedure that, when compared with the other methods, identified a short-form solution with the best fitting measurement model structure (modeled in a CFA), with the highest internal consistency and factor saturation (assessed with ω; Olaru et al., in press).

In line with the goals of this paper, ACO was specified to identify the shortest short-form solution that maintained an adequate omega estimate (ω = 0.70) and model fit in a CFA (*CFI* = 0.95; *RMSEA* = 0.02). A 10-item solution was identified as the shortest scale that matched both criteria (and in an additional 100 runs of the ACO, the same 10 items were identified over 90% of the time with deviating solutions of lesser psychometric quality; please see (Olaru et al., in press), for details on the procedure). In a CFA, all items loaded moderately to strongly on the latent factor. The average inter-item correlation (0.18) is acceptable according to Clark and Watson (1995), however the range of inter-item correlations (−0.07 to 0.36) still falls outside of their recommendations. At the latent level, this short-form solution was weakly related to both measures of Cognitive Empathy and moderately related to both Emotion Perception and Vocabulary. Because the ACO Model has an adequate omega estimate and adequate fit in a measurement model, we suggest the ACO Model is a sufficient short-form solution to the Eyes Test.

### Conclusion

In contrast to the complete Eyes Test, the ACO Model has adequate psychometric properties according to our criteria, suggesting this is a sufficient short-form solution to the complete Eyes Test. At the latent level, this solution was most related to Vocabulary, followed by Emotion Perception and Cognitive Empathy. Because the ACO solution might be somewhat overfitted on the current sample, we cross-validated this solution on a second sample.

## Study 2

### Methods

#### Participants

Participants were again sampled from Amazon Mechanical Turk. Initially, 233 persons participated, however after removing those with insufficient variance in their responses, our final sample was 210 persons (108 female), primarily White, non-Hispanic (82%; 11% Black, non-Hispanic; 3% Asian; 4% Other), on average 39.9 years of age (*SD* = 22.4), from a variety of education levels (9% High School or GED equivalent, 39% Some College, 52% Bachelor's Degree or higher). All participants were currently living in the USA (indicated by their IP addresses) and for 97% their native language was English.

#### Measures and procedure

We administered the same measures described in Study 1 (in order to reduce the testing time, we administered only those scales discussed in this paper) and the 10 ACO Model items of the Eyes Test. The internal consistency of all measures was acceptable: Perspective Taking (ω = 0.88); Emotion Specific Empathy Cognitive Empathy (ω = 0.94); DANVA 2 (ω = 0.95); Vocabulary (ω = 0.90).

### Results

The ACO Model solution in Study 2 had an adequate omega (ω = 0.73), indicating acceptable internal consistency and factor saturation. The average inter-item correlation (0.20) was acceptable according to Clark and Watson (1995), however the range of inter-item correlations (-0.07 to 0.59) still falls outside of their recommendation. The measurement model structure fit the data sufficiently according to the CFA fit statistics, with eight of the 10 items loading moderately to strongly on the latent factor. To test for measurement invariance in the scale structure between the two samples, we estimated a multiple-group analysis. Because the data is categorical, we applied a two-step approach recommended by Muthén and Muthén (2011). In the first model, comparable to traditional constraints associated with configural invariance, item thresholds and factor loadings were allowed to vary freely across the samples, while the scale factors were fixed at 1 and factor means were fixed at 0. Overall, this structure fit the data well according to all fit indices, which the exception of the WRMR [χ^2^_(70)_ = 79.29, *p* = 0.21, *RMSEA* = 0.020 _(0.000–0.038)_, *WRMR* = 1.082, *CFI* = 0.962, *TLI* = 0.951]. However, given that all fit indices, with the exception of the WRMR, indicate acceptable fit, we can ignore the WRMR in this instance (Muthén, 2014). Next, the item thresholds and factor loadings were constrained to be equal across samples, while the scale factors in Sample 1 were fixed to 1, the factor mean in Sample 1 was fixed to 0, and in Sample 2 the scale factors and factor mean was allowed to vary (considered similar to strong factorial invariance). This model also fit the data well [χ^2^_(79)_ = 85.31, *p* = 0.29, *RMSEA* = 0.015 _(0.000–0.035)_, *WRMR* = 1.160, *CFI* = 0.974, *TLI* = 0.971] and the constraints did not significantly decrease the fit of the model [Δχ^2^_(9)_ = 6.02, *p* = 0.74, estimated with the DIFFTEST option in MPlus] suggesting that overall, the ACO Model has equivalent structure in both samples.

At the latent level, the model was weakly to strongly related with measures of Cognitive Empathy, weakly related with Emotion Perception, and strongly related with Vocabulary. The relations with Emotion Perception, Vocabulary, and Perspective Taking were similar between Study 1 and Study 2, however the relation with ESE Cognitive Empathy was considerably higher. The change in relation could be due to differences in the loadings of indicators on their respective measurement models, difference in intercepts, and so forth, so the model was remodeled in a multiple-group analysis comparing both samples. Restrictions of configural invariance [χ^2^_(226)_ = 226.78, *p* = 0.15, *RMSEA* = 0.017 _(0.000–0.029)_, *WRMR* = 1.014, *CFI* = 0.990, *TLI* = 0.988] and strong factorial invariance [χ^2^_(226)_ = 241.41, *p* = 0.23, *RMSEA* = 0.014 _(0.000–0.027)_, *WRMR* = 1.130, *CFI* = 0.992, *TLI* = 0.992] fit the data well, and were not significantly different from one another [Δχ^2^_(20)_ = 14.63, *p* = 0.80].

To compare the latent correlation between the ACO Model and ESE Cognitive Empathy, we needed to create Phantom Variables, which control for differences in the latent variances between the two samples (Little, 1997). When we constrained the model so that the latent correlation between the two factors was equal across samples, the model still fit the data well [χ^2^_(227)_ = 264.04, *p* < 0.05, *RMSEA* = 0.022 _(0.003–0.032)_, *WRMR* = 1.253, *CFI* = 0.982, *TLI* = 0.981]. Model fit was, however, significantly worse [Δχ^2^_(1)_ = 22.63, *p* < 0.05]. Given that the final constrained model had acceptable fit, we will interpret the final constrained latent correlation (i.e., 0.460), as indicating overall a moderate relation between the ACO Model and ESE Cognitive Empathy.

### Conclusion

In a second sample, the ACO Model maintained an adequate omega estimate, indicating sufficient internal consistency and factor saturation, and demonstrable homogeneity. The model maintained moderate relations with Emotion Perception and strong relations with Vocabulary, and had weak to moderate relations with Cognitive Empathy. Overall, the adequacy of the ACO Model solution was supported in this second sample.

## Discussion

### Summary

We performed a psychometric test of the Reading the Mind in the Eyes Test and found, as reported by others (e.g., Harkness et al., 2010), that the complete test is not homogenous and has poor internal consistency. In previous work, several empirically derived short-form and subscale solutions were proposed. We evaluated these proposals on three criteria: (1) omega estimate, indicating internal consistency; (2) factor saturation; and (3) measurement model fit. Using these criteria, we identified a short-form solution (ACO Model) that meets both criteria. The ACO Model was sufficiently cross-validated in a second sample. At the latent level, the ACO Model is weakly to moderately related with Cognitive Empathy, moderately related with Emotion Perception, and has the strongest relation with Vocabulary.

Based on the results presented in this series of studies, and inferred from evidence by other groups also suggesting that the full measure has poor psychometric quality, we recommend the use of the 10-item ACO Model short-form solution instead of the full Eyes Test. This recommendation applies to all uses with unimpaired healthy adults where the goal is to assess individual differences in ToM. If the population or measurement intention changes, adequate evidence concerning the criteria we applied should be presented.

This short-form solution is homogeneous with adequate internal consistency suggesting it is a more precise measure of ToM compared with the original form. The short-form solution is considerably reduced from the complete Eyes Test, making it much quicker to administer. Also, the descriptive statistics suggest that the spread of person-level scores is comparable to that of the complete Eyes Test, thus the short-form solution still acts as an adequate individual difference measure.

Regarding the design, the ACO Model has an almost equal distribution of male and female identities, like the original version. Also, while it is possible that the ACO Model short-form solution might be conceptually limited from Baron-Cohen et al.'s (2001) original intentions, with an overuse of items where the target item is neutral or negative (based on the affect classification provided by Harkness et al., 2005), we argue that this is not the case. First, Baron-Cohen and colleagues did not discuss purposely including items with different affects and they never proposed that the affect of the target word could be used to create subscales or should bias performance on the test, so in our analyses we operated under the assumption that there was no preference in terms of which items were the most appropriate for the measure of ToM. Also, as presented above, we found that the affect classifications by Konrath et al. (2014) and by Harkness et al. (2005) were a poor organization of the items, suggesting that the affect of the target word does not predict performance in any meaningful way. Thus, the ACO short-form solution should not be considered to be a measure of ToM that is biased toward assessing ToM of negative mental states any more than that assumption is made of the original test.

### Convergent validity evidence

The moderate latent relations with Cognitive Empathy and Emotion Perception were expected based on Baron-Cohen et al. (2001) intentions and based on previous literature. ToM is largely considered by some to be similar to, if not the same as, Cognitive Empathy (e.g., Lawrence et al., 2004). That these two were not more strongly correlated can be attributed to the differences in measurement methodologies (Nunnally and Bernstein, 1994). Likewise, the moderate relation with Emotion Perception is also supported in the literature (e.g., Henry et al., 2009). Both ToM and Emotion Perception tests are described as ability tests and both test the extent to which participants can read the minds, facial expressions, and feelings of another person. Given these overlaps, it should be considered that ToM, as measured with the Eyes Test, is part of a larger set of research that includes Emotion Perception, referred to as social intelligence, emotional intelligence, interpersonal abilities, and/or socio-emotional abilities.

Finally, while a relation with Vocabulary was expected, we did not expect such a strong relation. Previous research suggests that at the manifest level, the Eyes Test is weakly related to verbal fluency (Ahmed and Miller, 2011) and moderately related to verbal IQ (Golan et al., 2006; Peterson and Miller, 2012). At the latent level, however, we found a strong correlation (0.49 in Study 1 and 0.62 in Study 2) between the ACO Model with Vocabulary, which is similar in magnitude to the manifest correlation with Vocabulary found by Peterson and Miller (2012). That this is the second study to identify such a strong relation between the Eyes Test and Vocabulary suggests that performance on the former may be heavily based on one's vocabulary knowledge. The response options of the Eyes Test, in general, occur less frequently in the English language [e.g., the mean frequency of the response options, based on the Corpus of Contemporary American English (Davies, 2008), is 8556] compared with other descriptive terms [e.g., the six based emotions (anger, disgust, fear, happy, sad, surprise) where the mean frequency is 28,435]. In fact, based on our Study 1 data, the frequency of the target word is weakly correlated (*r* = 0.10) with how often that response option was selected by the participants meaning target words that occur more frequently were more likely to be correctly chosen by the participants. The test's reliance on Vocabulary was mentioned by the test designers, however the inclusion of the response option definitions in the instructions for the test was designed to mitigate this effect. Our results, and that of others, suggests this may not be sufficient. Future revisions of the Eyes Test should focus on minimizing the test's reliance on Vocabulary, so that the test might be a more precise measure of ToM and less a measure of Vocabulary.

## Conclusions

Overall, we were able to identify an adequate short-form solution to the Eyes Test that is homogeneous and has adequate internal consistency. That short-form solution was strongly related to Vocabulary, moderately related to Emotion Perception, and moderately to weakly related with self-reported Cognitive Empathy. Given the relatively high relation with Emotion Perception, and other research that consistently identifies the Eyes Test as related to Emotion Perception, and sometimes a measure of that construct, future research should attempt to incorporate ToM research, in particular the Eyes Test, in research on emotional intelligence and interpersonal abilities with the goal of identifying the convergent and discriminant validity of this test amongst these abilities. Finally, the strong relation with Vocabulary suggests that the Eyes Test still has a large reliance on one's vocabulary, and future revisions of the test should try to reduce that bias.

The relations of the Eyes Test with Emotion Perception and Vocabulary is of particular importance for the study of clinical populations identified to have a deficit in ToM. Given that the Eyes Test correlates moderately with Emotion Perception and strongly with Vocabulary, as the Eyes Test is designed currently one cannot rule out the possibility that perhaps the Eyes Test discriminates between these groups on Emotion Perception and Vocabulary, and not just on ToM. Research regarding the extent to which individuals on the Autism Spectrum have a deficit in their ability to perceive emotions in the face (cf. Harms et al., 2010) and a deficit in language ability (cf. Bosseler and Massaro, 2003) is mixed, however findings generally point to a deficit in both. Thus, the differences in groups identified by the Eyes Test might be because the test identifies differences between these groups in their Vocabulary and Emotion Perception abilities. This should be examined in future research.

### Conflict of interest statement

The authors declare that the research was conducted in the absence of any commercial or financial relationships that could be construed as a potential conflict of interest.

## Footnotes

^1^High School, GED equivalent, or Less than High School (*n* = 65, *M* = 26.54, *SD* = 3.64); Some College (*n* = 159, *M* = 27.10, *SD* = 3.75); Associates Degree (*n* = 54, *M* = 27.41, *SD* = 4.16); Bachelors Degree (*n* = 168, *M* = 27.61, *SD* = 3.72); More than a Bachelors Degree (e.g., Masters, MD, JD, PhD) (*n* = 38, *M* = 26.68, *SD* = 4.22).

^2^Females: *M* = 27.43, *SD* = 3.74; males: *M* = 26.98, *SD* = 3.89.
