# Supplementary material for: A psychometric analysis of the reading the mind in the eyes test: toward a brief form for research and applied settings
Source: Front Psychol. 2015 Oct 6;6:1503. doi: 10.3389/fpsyg.2015.01503 (PMC4593947; doi:10.3389/fpsyg.2015.01503)
Supplement: Supplementary file 1 [file DataSheet1.DOCX]

**Appendix A1**

*Study 1:* *Relative Frequency of Each Response Option for Each Item of the Eyes Test*

| **Item** | | **Target Word** | | **Foil 1** | | **Foil 2** | | **Foil 3** | |
| --- | --- | --- | --- | --- | --- | --- | --- | --- | --- |
| **#** | **Stimuli Sex** | **Response Option** | **Relative Frequency** | **Response Option** | **Relative Frequency** | **Response Option** | **Relative Frequency** | **Response Option** | **Relative Frequency** |
| 1 | Male | **Playful** | .70 | Comforting | .09 | **Irritated** | .18 | **Bored** | .02 |
| 2 | Male | Upset | .75 | **Terrified** | .14 | **Arrogant** | .01 | **Annoyed** | .10 |
| 3 | Female | Desire | .89 | **Joking** | .02 | **Flustered** | .03 | Convinced | .06 |
| 4 | Male | **Insisting** | .73 | **Joking** | .02 | **Amused** | .06 | Relaxed | .19 |
| 5 | Male | Worried | .91 | **Irritated** | .04 | **Sarcastic** | .05 | **Friendly** | .01 |
| 6 | Female | **Fantasizing** | .88 | **Aghast** | .02 | **Impatient** | .09 | **Alarmed** | .02 |
| 7 | Male | Uneasy | .65 | **Apologetic** | .05 | **Friendly** | .24 | **Dispirited** | .06 |
| 8 | Male | **Despondent** | .89 | Relieved | .07 | **Shy** | .03 | **Excited** | .01 |
| 9 | Female | **Preoccupied** | .90 | **Annoyed** | .06 | **Hostile** | .01 | **Horrified** | .03 |
| 10 | Male | **Cautious** | .58 | **Insisting** | .28 | **Bored** | .10 | **Aghast** | .03 |
| 11 | Male | Regretful | .69 | **Terrified** | .19 | **Amused** | .10 | **Flirtatious** | .02 |
| 12 | Male | Skeptical | .88 | Indifferent | .10 | **Embarrassed** | .02 | **Dispirited** | .01 |
| 13 | Male | Anticipating | .82 | **Decisive** | .05 | Threatening | .03 | **Shy** | .10 |
| 14 | Male | Accusing | .83 | **Irritated** | .09 | **Disappointed** | .04 | Depressed | .04 |
| 15 | Female | **Contemplative** | .74 | **Flustered** | .04 | **Encouraging** | .09 | **Amused** | .14 |
| 16 | Male | Thoughtful | .81 | **Irritated** | .05 | **Encouraging** | .05 | Sympathetic | .10 |
| 17 | Female | Doubtful | .38 | **Affectionate** | .38 | **Playful** | .20 | **Aghast** | .03 |
| 18 | Female | **Decisive** | .62 | **Amused** | .23 | **Aghast** | .06 | **Bored** | .09 |
| 19 | Female | Tentative | .61 | **Arrogant** | .05 | **Grateful** | .23 | **Sarcastic** | .11 |
| 20 | Male | **Friendly** | .91 | Dominant | .05 | **Guilty** | .04 | **Horrified** | .004 |
| 21 | Female | **Fantasizing** | .91 | **Embarrassed** | .05 | Confused | .03 | **Panicked** | .004 |
| 22 | Female | **Preoccupied** | .81 | **Grateful** | .02 | **Insisting** | .05 | Imploring | .13 |
| 23 | Male | Defiant | .53 | **Contented** | .10 | **Apologetic** | .07 | Curious | .30 |
| 24 | Male | Pensive | .76 | **Irritated** | .13 | **Excited** | .03 | **Hostile** | .08 |
| 25 | Female | **Interested** | .68 | **Panicked** | .05 | Incredulous | .21 | **Despondent** | .07 |
| 26 | Male | **Hostile** | .77 | **Alarmed** | .06 | **Shy** | .06 | Anxious | .11 |
| 27 | Female | **Cautious** | .80 | **Joking** | .02 | **Arrogant** | .07 | Reassuring | .11 |
| 28 | Female | **Interested** | .66 | **Joking** | .02 | **Affectionate** | .26 | **Contented** | .06 |
| 29 | Female | Reflective | .78 | **Impatient** | .10 | **Aghast** | .02 | **Irritated** | .11 |
| 30 | Female | **Flirtatious** | .94 | **Grateful** | .01 | **Hostile** | .03 | **Disappointed** | .01 |
| 31 | Female | Confident | .71 | **Ashamed** | .05 | **Joking** | .07 | **Dispirited** | .17 |
| 32 | Male | Serious | .78 | **Ashamed** | .03 | Bewildered | .11 | **Alarmed** | .08 |
| 33 | Male | Concerned | .74 | **Embarrassed** | .03 | **Guilty** | .16 | **Fantasizing** | .08 |
| 34 | Female | Distrustful | .68 | **Aghast** | .05 | Baffled | .21 | **Terrified** | .05 |
| 35 | Female | **Nervous** | .59 | Puzzled | .13 | **Insisting** | .15 | **Contemplative** | .13 |
| 36 | Male | Suspicious | .91 | **Ashamed** | .01 | **Nervous** | .01 | Indecisive | .06 |

*Note.* Bolding indicates words were included as a response option more than once.

**Appendix A2**

*Study 1:* *Tetrachoric Correlations Between All Items of the Eyes Test*

|  | #1 | #2 | #3 | #4 | #5 | #6 | #7 | #8 | #9 | #10 | #11 | #12 | #13 | #14 | #15 | #16 | #17 | #18 | #19 | #20 | #21 | #22 | #23 | #24 | #25 | #26 | #27 | #28 | #29 | #30 | #31 | #32 | #33 | #34 | #35 |  |
| --- | --- | --- | --- | --- | --- | --- | --- | --- | --- | --- | --- | --- | --- | --- | --- | --- | --- | --- | --- | --- | --- | --- | --- | --- | --- | --- | --- | --- | --- | --- | --- | --- | --- | --- | --- | --- |
| #2 | .04 |  |  |  |  |  |  |  |  |  |  |  |  |  |  |  |  |  |  |  |  |  |  |  |  |  |  |  |  |  |  |  |  |  |  |  |
| #3 | -.15 | .22 |  |  |  |  |  |  |  |  |  |  |  |  |  |  |  |  |  |  |  |  |  |  |  |  |  |  |  |  |  |  |  |  |  |  |
| #4 | -.07 | .10 | .16 |  |  |  |  |  |  |  |  |  |  |  |  |  |  |  |  |  |  |  |  |  |  |  |  |  |  |  |  |  |  |  |  |  |
| #5 | .03 | .13 | .07 | .03 |  |  |  |  |  |  |  |  |  |  |  |  |  |  |  |  |  |  |  |  |  |  |  |  |  |  |  |  |  |  |  |  |
| #6 | .23 | .06 | .17 | -.07 | .31 |  |  |  |  |  |  |  |  |  |  |  |  |  |  |  |  |  |  |  |  |  |  |  |  |  |  |  |  |  |  |  |
| #7 | -.04 | -.05 | .02 | .16 | .07 | -.06 |  |  |  |  |  |  |  |  |  |  |  |  |  |  |  |  |  |  |  |  |  |  |  |  |  |  |  |  |  |  |
| #8 | .03 | .05 | .07 | .17 | .12 | .11 | .12 |  |  |  |  |  |  |  |  |  |  |  |  |  |  |  |  |  |  |  |  |  |  |  |  |  |  |  |  |  |
| #9 | -.03 | .01 | .00 | -.14 | -.03 | .01 | -.02 | .05 |  |  |  |  |  |  |  |  |  |  |  |  |  |  |  |  |  |  |  |  |  |  |  |  |  |  |  |  |
| #10 | -.01 | .01 | .10 | .18 | .14 | -.02 | .27 | .21 | .01 |  |  |  |  |  |  |  |  |  |  |  |  |  |  |  |  |  |  |  |  |  |  |  |  |  |  |  |
| #11 | .08 | .11 | .23 | -.01 | .10 | -.05 | -.01 | .18 | .05 | .09 |  |  |  |  |  |  |  |  |  |  |  |  |  |  |  |  |  |  |  |  |  |  |  |  |  |  |
| #12 | .08 | .04 | .04 | .16 | -.09 | -.12 | .15 | .25 | .02 | .00 | -.03 |  |  |  |  |  |  |  |  |  |  |  |  |  |  |  |  |  |  |  |  |  |  |  |  |  |
| #13 | .10 | .13 | .02 | .00 | .08 | .14 | -.01 | .22 | .14 | -.08 | .16 | -.03 |  |  |  |  |  |  |  |  |  |  |  |  |  |  |  |  |  |  |  |  |  |  |  |  |
| #14 | .16 | .16 | .16 | .17 | .14 | -.06 | -.10 | .28 | .27 | .08 | .02 | .34 | .10 |  |  |  |  |  |  |  |  |  |  |  |  |  |  |  |  |  |  |  |  |  |  |  |
| #15 | .01 | .10 | .08 | .12 | .04 | .01 | .06 | .32 | .29 | .18 | .19 | .25 | -.01 | .10 |  |  |  |  |  |  |  |  |  |  |  |  |  |  |  |  |  |  |  |  |  |  |
| #16 | .08 | .04 | -.13 | .06 | .10 | -.14 | .05 | .10 | .33 | -.03 | -.06 | .10 | .08 | .14 | .17 |  |  |  |  |  |  |  |  |  |  |  |  |  |  |  |  |  |  |  |  |  |
| #17 | .14 | -.14 | .00 | .14 | .01 | -.08 | .08 | .17 | .03 | .17 | .03 | -.07 | -.04 | .03 | .10 | .07 |  |  |  |  |  |  |  |  |  |  |  |  |  |  |  |  |  |  |  |  |
| #18 | .10 | -.01 | .12 | .19 | -.04 | -.02 | .10 | .07 | .00 | .22 | .07 | .08 | -.08 | .04 | .12 | .07 | .16 |  |  |  |  |  |  |  |  |  |  |  |  |  |  |  |  |  |  |  |
| #19 | .05 | -.10 | .11 | .06 | .17 | -.09 | .08 | .30 | .29 | .18 | .10 | .14 | .05 | .17 | .20 | .17 | .08 | -.02 |  |  |  |  |  |  |  |  |  |  |  |  |  |  |  |  |  |  |
| #20 | .21 | .21 | -.13 | .04 | -.01 | .09 | .02 | .07 | .28 | .21 | .05 | -.02 | .00 | .19 | .06 | .11 | .10 | .20 | -.07 |  |  |  |  |  |  |  |  |  |  |  |  |  |  |  |  |  |
| #21 | .11 | .11 | .29 | .04 | .13 | .28 | .08 | .01 | .17 | -.04 | .18 | -.09 | .17 | -.28 | .06 | .02 | -.26 | .07 | -.14 | .14 |  |  |  |  |  |  |  |  |  |  |  |  |  |  |  |  |
| #22 | .05 | .06 | .04 | .04 | -.13 | -.02 | -.03 | .35 | .16 | -.05 | .13 | -.04 | .05 | .11 | .21 | .00 | .05 | .22 | .05 | .19 | .11 |  |  |  |  |  |  |  |  |  |  |  |  |  |  |  |
| #23 | .14 | -.01 | .15 | .24 | -.07 | -.08 | .07 | .04 | .00 | .02 | .14 | .12 | .02 | .17 | .10 | .00 | .09 | .20 | .09 | -.12 | -.12 | .25 |  |  |  |  |  |  |  |  |  |  |  |  |  |  |
| #24 | .03 | .15 | .08 | .09 | .29 | .02 | .26 | .36 | .17 | .26 | .07 | .18 | -.01 | .19 | .07 | .15 | -.02 | .12 | .11 | .05 | .01 | .15 | .06 |  |  |  |  |  |  |  |  |  |  |  |  |  |
| #25 | .14 | .04 | .32 | -.22 | .01 | .18 | -.10 | -.11 | .18 | -.09 | -.03 | -.11 | .13 | .17 | .00 | .00 | -.11 | .03 | .11 | .13 | -.01 | .08 | -.07 | .07 |  |  |  |  |  |  |  |  |  |  |  |  |
| #26 | .07 | .05 | .21 | .14 | .22 | .01 | .04 | .03 | .05 | .01 | .19 | .15 | -.14 | .13 | -.02 | .09 | .15 | .00 | -.07 | .16 | -.04 | -.03 | .00 | .01 | .04 |  |  |  |  |  |  |  |  |  |  |  |
| #27 | .16 | .06 | .07 | .09 | .00 | .00 | .25 | .05 | .11 | .08 | .01 | -.06 | .12 | .04 | .13 | .12 | .02 | -.01 | .07 | .10 | .01 | .07 | .18 | .13 | .02 | .05 |  |  |  |  |  |  |  |  |  |  |
| #28 | .15 | .01 | .10 | .08 | -.01 | -.07 | .27 | .24 | .04 | -.03 | .07 | .07 | .18 | .20 | .14 | .23 | .19 | .12 | .12 | .17 | .23 | .25 | .18 | .13 | .06 | .05 | .31 |  |  |  |  |  |  |  |  |  |
| #29 | .14 | .06 | .12 | -.05 | -.04 | .21 | -.06 | .10 | .13 | .02 | .14 | .10 | .05 | .14 | .27 | -.05 | -.12 | .03 | .09 | .05 | .01 | .08 | .04 | .00 | .13 | -.04 | .09 | -.03 |  |  |  |  |  |  |  |  |
| #30 | -.01 | .29 | .41 | .02 | -.22 | .12 | -.14 | -.12 | -.08 | -.03 | .07 | -.03 | -.01 | -.15 | .14 | -.10 | -.19 | -.03 | .09 | -.06 | .14 | -.10 | -.05 | .17 | .31 | .14 | -.04 | .02 | -.08 |  |  |  |  |  |  |  |
| #31 | .09 | -.12 | .07 | .01 | .21 | .22 | -.11 | .11 | .09 | .05 | .12 | .01 | -.16 | .04 | -.03 | .18 | -.23 | .03 | .00 | .05 | .19 | -.04 | -.01 | -.03 | .05 | .03 | -.01 | .01 | .06 | -.06 |  |  |  |  |  |  |
| #32 | .07 | .04 | -.10 | .08 | .16 | .02 | .01 | .20 | .24 | .01 | .15 | .20 | .08 | .21 | .20 | .09 | -.03 | .28 | .11 | .13 | .13 | .18 | .06 | .19 | .14 | -.05 | -.03 | .13 | -.02 | .10 | .20 |  |  |  |  |  |
| #33 | .16 | .27 | -.10 | -.05 | -.03 | .12 | -.05 | .24 | .02 | .08 | .09 | .08 | .17 | .18 | .16 | .16 | .14 | .18 | .15 | .05 | -.02 | .18 | .00 | .04 | .15 | .06 | .12 | .16 | .09 | .03 | .02 | .14 |  |  |  |  |
| #34 | .00 | .14 | .22 | .14 | .18 | .16 | .15 | .08 | -.07 | .20 | .14 | -.02 | .01 | .07 | .13 | .07 | .07 | .13 | .21 | .07 | .10 | .23 | -.07 | .26 | .09 | .11 | .11 | .10 | .07 | .05 | -.03 | .07 | -.04 |  |  |  |
| #35 | .14 | .08 | .11 | .05 | .24 | -.01 | .13 | .16 | .02 | .17 | .29 | .01 | .11 | .07 | .10 | .13 | .00 | .22 | .15 | -.17 | .10 | .11 | .25 | .20 | -.03 | -.02 | .09 | .10 | .03 | -.12 | -.03 | .08 | .03 | .34 |  |  |
| #36 | -.15 | -.08 | .20 | -.03 | .07 | .10 | .03 | .13 | .33 | .07 | .02 | .05 | .14 | .24 | -.01 | -.01 | .02 | .02 | .30 | .08 | .01 | .12 | .22 | .06 | .20 | -.08 | .15 | .25 | -.12 | .14 | .17 | -.07 | .10 | .01 | .24 |  |

*Note.* These were estimated to the fourth decimal place and used in that manner for analyses. Due to space limitations, the values here are truncated.
